# Identification of Novel Small Molecules as Inhibitors of Hepatitis C Virus by Structure-Based Virtual Screening

**DOI:** 10.3390/ijms141122845

**Published:** 2013-11-20

**Authors:** Jing Li, Xian Liu, Shanshan Li, Yulan Wang, Nannan Zhou, Cheng Luo, Xiaomin Luo, Mingyue Zheng, Hualiang Jiang, Kaixian Chen

**Affiliations:** 1 State Key Laboratory of Drug Research, Shanghai Institute of Materia Medica, Chinese Academy of Sciences, 555 Zuchongzhi Road, Shanghai 201203, China; E-Mails: jingli@mail.shcnc.ac.cn (J.L.); xliu@maill.shcnc.ac.cn (X. Liu); simlink_lishanshan@163.com (S.L.); wangylworld@163.com (Y.W.); cluo@maill.shcnc.ac.cn (C.L.); xmluo@maill.shcnc.ac.cn (X. Luo); hljiang@maill.shcnc.ac.cn (H.J.); kxchen@maill.shcnc.ac.cn (K.C.); 2 State Key Laboratory of Bioreactor Engineering and Shanghai Key Laboratory of Chemical Biology, School of Pharmacy, East China University of Science and Technology, Shanghai 200237, China; E-Mail: znn0912@gmail.com

**Keywords:** hepatitis C virus (HCV), NS3/NS4A serine protease, structure-based drug design (SBDD), virtual screening

## Abstract

Hepatitis C virus (HCV) NS3/NS4A serine protease is essential for viral replication, which is regarded as a promising drug target for developing direct-acting anti-HCV agents. In this study, sixteen novel compounds with cell-based HCV replicon activity ranging from 3.0 to 28.2 μM (IC_50_) were successfully identified by means of structure-based virtual screening. Compound **5** and compound **11**, with an IC_50_ of 3.0 μM and 5.1 μM, respectively, are the two most potent molecules with low cytotoxicity.

## 1. Introduction

Hepatitis C virus (HCV) is a disease of global concern, which has infected about 170 million people worldwide and caused serious liver diseases, such as cirrhosis and hepatocellular carcinoma [1,2]. The high variability of virus leads to the formation of different HCV genotypes, and the predominant genotype of type 1 accounts for more than 60% of infections [3]. The current standard-of-care therapy for HCV infected patients only shows about 45% sustained viral responses (SVR) in HCV genotype 1-infected people [4,5]. Clearly, the ineffectiveness of viral DNA elimination in such a large population of patients triggers enhanced investigations towards direct-acting antiviral drugs (DAAs) for hepatitis C. Currently, several promising targets for HCV have emerged, including NS3/NS4A protease and the NS5B RNA-dependent RNA polymerase [6,7].

**Figure 1:**
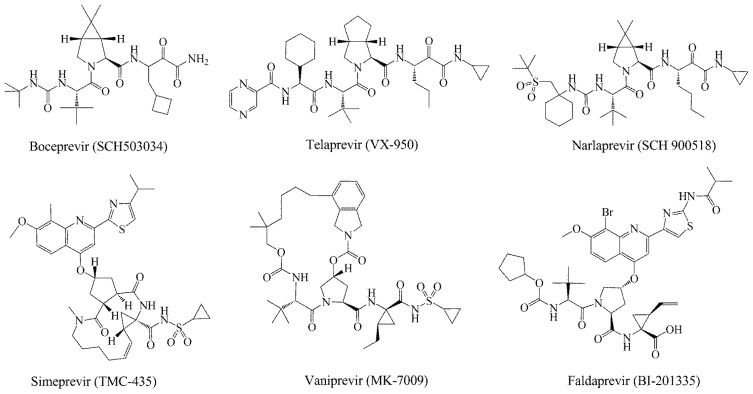
Some potent hepatitis C virus (HCV) NS3/NS4A serine protease inhibitors under clinical trials or in the market.

HCV non-structure (NS) proteins, NS3/NS4A serine protease, have been intensively studied as an attractive drug target for the development of DAAs [8]. The *N*-terminal domain of non-structural protein NS3 shows protease activity in complex with cofactor NS4A. The complex catalyzes the proteolytic cleavage of HCV polyprotein at four junctions between NS3, NS4A, NS4B, NS5A and NS5B and is hence responsible for the maturation of HCV polyprotein [9,10]. Nowadays, several inhibitors of NS3 protease have been reported in the literature, mainly explored for the optimization of natural substrates [2,11–15]. Additionally, some of the peptidomimetics endowed with high potential are being studied in clinical trials, of which the most advanced are listed in Figure 1. These inhibitors can be classified into two types depending on their mechanism of action: (1) reversible covalent inhibitors form a covalent bond with a residue of the catalytic triad (Ser139), such as linear α-ketoamide (SCH503034, VX-950 and SCH900518); and (2) non-covalent inhibitors depend on the *C*-terminal carboxylate (TMC-435, MK-7009 and BI201335), which is mostly a macrocyclic compound [16–21]. Among them, Telaprevir [22] and Boceprevir [23] have been approved by the FDA (U.S. Food and Drug Administration) as the therapy of chronic HCV genotype 1 in May, 2011.

Although these peptidomimetic inhibitors show potent activity against NS3/NS4A serine protease, their relatively high molecular weight and poor chemical stability lead to a problematic ADME (absorption, distribution, metabolism and excretion) profile [24]. In addition, various mutation sites were reported to confer resistance to these protease inhibitors, such as the broad cross-resistant mutation sites, R155K and A156T/S, for all protease inhibitors and the D168A/V mutation, usually associated with macrocyclic inhibitors [25,26]. Structural diversity is significant for developing the next generation HCV protease inhibitors to combat resistance [27]. Therefore, it is a major challenge to discover and develop novel small compounds to overcome viral resistance mutation and exhibit better pharmacokinetic properties.

Structure-based drug design (SBDD) is a very robust and useful approach for hit identification. So far, several crystal structures of various ligand-bound HCV NS3/NS4A proteases have been determined, which provide insights into the inhibitor binding modes and also allowed the SBDD of novel HCV inhibitors. Based on the crystal structure of HCV NS3/NS4A protease, many computational approaches have been used in the binding model analysis and structure modifications for more potent HCV NS3/NS4A protease inhibitors [28–31]. Furthermore, several theoretical studies have been investigated on the reaction mechanism of the NS3/NS4A protease with natural substrates, mainly via quantum mechanics/molecular mechanic (QM/MM) calculation [32,33]. Herein, we report the discovery of some small non-peptide inhibitors via structure-based virtual screening, followed by HCV replicon cell assay and NS3/NS4A protease assay.

## 2. Results and Discussion

### 2.1. Structure-Based Virtual Screening of SPECS Database

**Figure 2:**
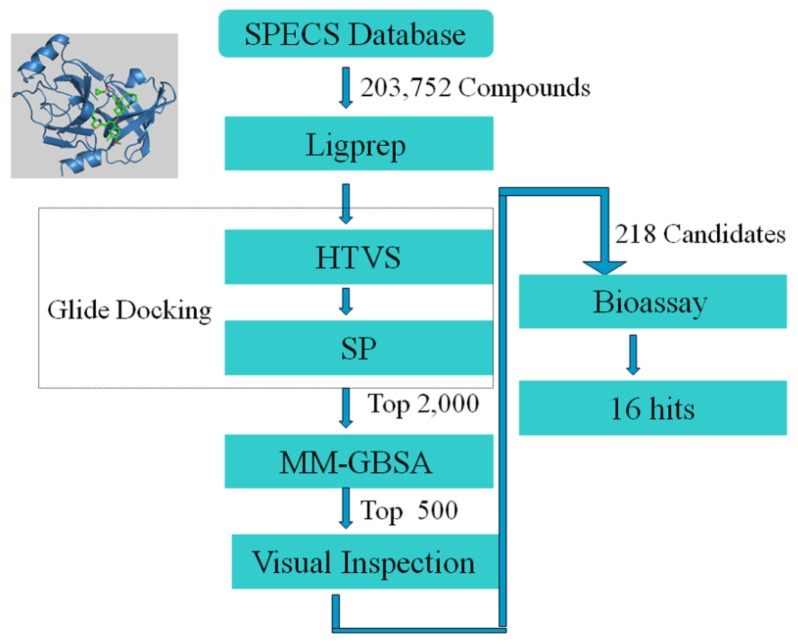
A workflow describing the multistep strategy as implemented in hit discovery for HCV NS3/NS4A protease. HTVS, high throughput virtual screening; SP, standard precision; MM-GBSA, molecular mechanic/generalized born surface area.

To obtain novel small molecule inhibitors of HCV NS3/NS4A protease, we designed a multistep virtual screening protocol. As illustrated in Figure 2, the commercial compound database, SPECS, was virtually screened with the molecular docking and the MM-GBSA (molecular mechanic/generalized born surface area) rescoring. Then, the candidate molecules were selected by comparing their binding modes with that of the noncovalent inhibitor Simeprevir (TMC-435). Finally, 218 candidate molecules were selected and purchased from SPECS and then evaluated in the HCV replicon system.

### 2.2. *In Vitro* Evaluation of the Selected Compounds

The 218 molecules purchased from SPECS were evaluated by cell-based HCV replicon assay and NS3/NS4A protease assay. This subgenomic replicon system developed by Lohmann *et al*. was used to perform bioactivity-guided screening to explore compounds with any anti-HCV activity [34,35]. Meanwhile, cyclosporine A (CSA) was tested as a positive control. Sixteen hits were proven to present dose-dependent inhibition activity toward the HCV replicon with the half-maximum inhibitory concentration (IC_50_) values less than 30 μM, which include ten molecules containing an *N*-acylhydrazone (RCONHN=CHR) moiety in common (Table 1) and another six compounds with different scaffolds (Table 2). A systematical literature survey revealed that these sixteen compounds have not been previously reported with any anti-HCV activities, but some of them share a common fragment with known HCV NS3/NS4A protease inhibitors. Recently, Takaya *et al.* [31] reported a series of NS3/NS4A inhibitors with a scaffold featuring *N*-acylhydrazone and a biaryl ester. Compared with these compounds, our hits provide more diverse substitutions at sites R_1_ and R_2_ and, more importantly, do not require possessing a biaryl ester. As shown in Table 1, a common structural feature shared by these analogs is the substituted hydroxylphenyl group at the R_2_ position. Meanwhile, the R_1_ substituted groups are relatively diverse. Among the ten screened compounds, four compounds, including **2**–**4** and **9**, showed a replicon inhibition IC_50_ of more than 10 μM, six compounds, **1**, **5**–**8** and **10**, showed higher activities at the micro molar level. From the six compounds of diverse scaffolds shown in Table 2, only two molecules displayed inhibition activities less than 10 μM, including compound **11**, showing the second highest potency among all the active compounds.

**Figure 3:**
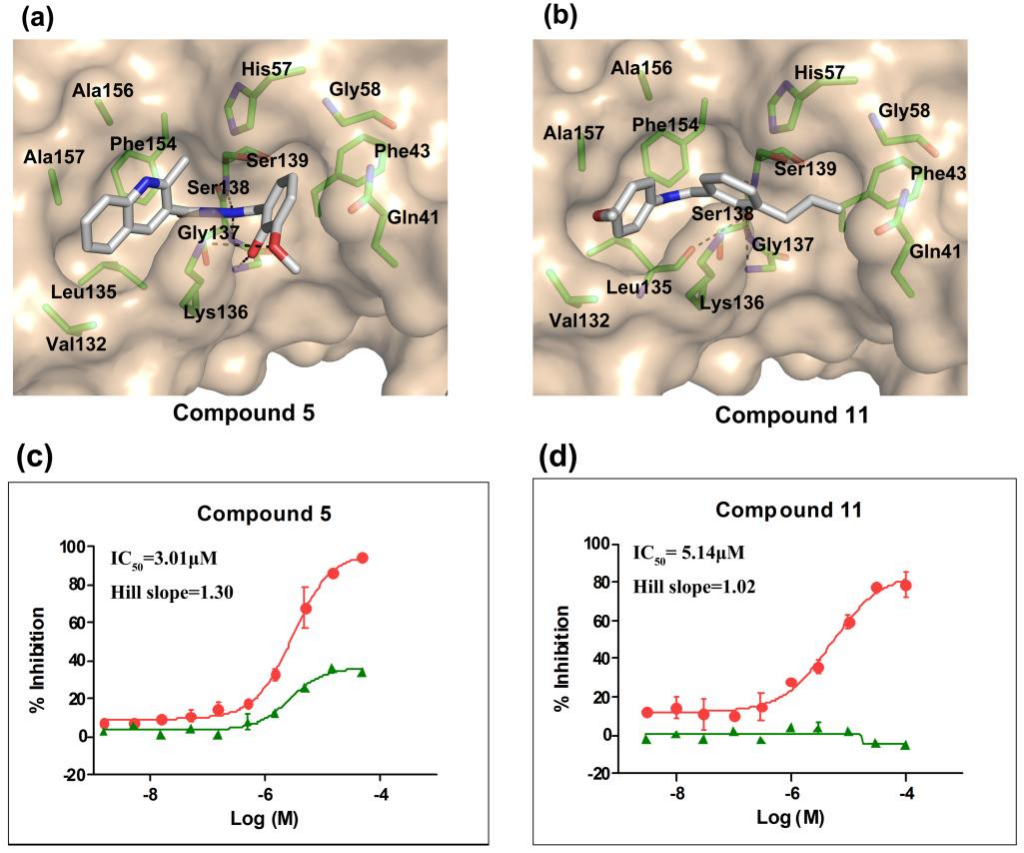
The binding models and dose-response curves of compound **5** (**a**,**c**) and compound **11** (**b**,**d**). (**a**) Glide-predicted binding pose for compound **5** within the active site of HCV NS3/NS4A serine protease (PDB ID: 3KEE); (**b**) glide-predicted binding pose for compound **11**; (**c**) dose-response curve for compound **5** (circular dots) determined using HCV replicon system; (**d**) dose-response curve for compound **11** (circular dots) determined using HCV replicon system. Cyclosporine A (CSA) was used as a positive control (green triangles).

According to the bioassay results summarized in Tables 1 and 2, compounds **5** and **11** are the two most active hits, exhibiting more than a nine-fold selectivity over cytotoxicity (CC_50_ > 50 μM) measured by a MTT colorimetric assay, with replicon assay IC_50_ values of 3.0 μM and 5.1 μM, respectively. The dose-response curves of compound **5** and **11** are depicted in Figure 3. Compound **5** is an *N*-acylhydrazone analog, which demonstrated a better selectivity index and replicon activity than the most potent analog (CP3-3284-53) reported by Takaya *et al*. [31]. Remarkably, the *N*-acylhydrazone moiety has been considered as an attractive privileged structure in some cysteine protease inhibitors, such as cruzipain and trophozoite cysteine protease inhibitors [36–38]. The current study highlighted that *N*-acylhydrazone is also a promising scaffold for developing HCV NS3/NS4A protease inhibitors.

**Figure 4:**
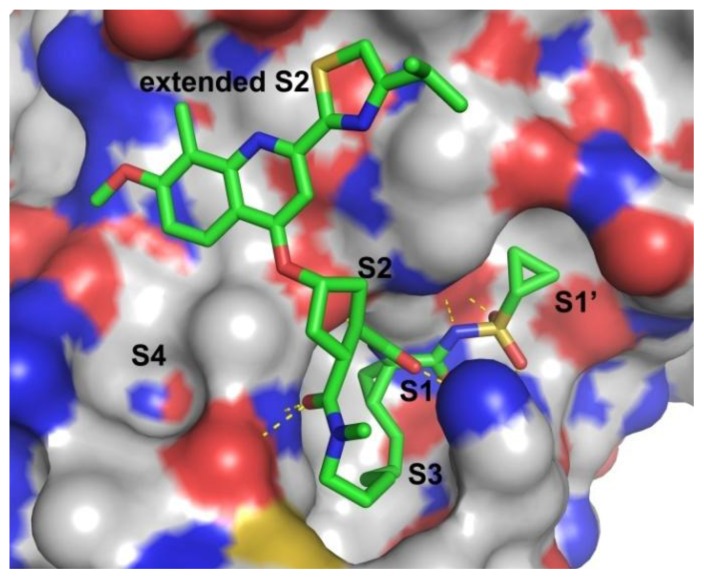
Crystal structure of HCV NS3/NS4A protease in complex with TMC-435 (PDB ID: 3KEE) [19]. Binding subsites of S1’-S4 in the active site are indicated on the surface representation and labeled in black. The bound inhibitor, TMC-435, is shown as a ball-and-stick model and is colored by atom type. For clarity, hydrogen atoms are omitted.

Figure 4 shows the protein surface with substrate binding subsites labeled [39]. Analysis of the X-ray structure of the NS3/NS4A protease revealed an expected trypsin-like fold for the enzyme, with a shallow and solvent-exposed substrate binding groove [9]. Accordingly, the binding affinity of NS3/NS4A protease inhibitors can be attributed to an extensive network of hydrogen bonds and lipophilic interactions [40]. The X-ray structure of NS3/NS4A protease demonstrates that the S1 and S3 subsites are in close proximity and characterized as hydrophobic pockets, which are lined with the hydrophobic residues of Val132, Leu135, Phe154, Ala156 and Ala157. Many potent P1–P3 macrocyclic inhibitors have been reported occupying these two pockets [9,13,39]. For further inhibition mechanism exploration, molecular modeling studies were also presented to reveal the binding interactions between ligand and HCV protease, which could provide the molecular basis for further lead optimization and structure-activity relationship analysis.

### 2.3. Molecular Modeling of the Hit Compounds

To investigate how the hit compounds interact with HCV NS3/NS4A protease, the predicted binding modes of the two most potent compounds (**5** and **11**, Tables 1 and 2) were shown in Figure 3. It can be found that these two compounds, despite possessing different scaffolds, share a similar pattern in forming two or more hydrogen bonds in the S1 subsite, *i.e*., the catalytic region. This interaction pattern has also been observed in previous macrocyclic noncovalent inhibitors binding to the active site of NS3/NS4A crystal complexes [19], as depicted in Figure 4. The extensive hydrogen bonding network formed in this region involves Ser139 of the catalytic triad and the secondary amine group (–NH–) of Gly137 and Ser138 in the oxyanion hole. For the compounds listed in Table 1, this region corresponds to the R2 moiety. For example, the R2 of compound **5** forms hydrogen bonds with Lys136 and Gly137, which is an ortho- and meta-substituted phenyl group. As shown in Table 1, the only structural difference between compound **8** and **9** is that the phenyl group at the R2 position is ortho- and meta-dihydroxyl substituted in **8**, but meta- and para-dihydroxyl substituted in **9**. Since the IC_50_ value of compound **8** is three-fold lower than compound **9**, we may infer that the hydroxyl substitution at the ortho- or meta-position is more favorable in forming hydrogen bonds within this site. From the crystal structure shown in Figure 4, we may notice that a cyclopropyl group of TMC-435 occupies the S1’ subsite of NS3/NS4A, formed by the residues of Gln41, Phe43 and Gly58. In a similar vein, an allyl group in compound **11** is located in the S1’ pocket (Figure 3b), which could mimic the interaction of the cyclopropyl moiety of TMC-435 [40].

## 3. Computational and Experimental Methods

### 3.1. *In Silico* Experiment Schema

The crystal structure of HCV NS3/NS4A serine protease in complex with a noncovalent inhibitor, TMC-435 (PDB entry: 3KEE; genotype 1b) [17], was used for a docking study. It is the first noncovalent NS3/NS4A protease-inhibitor crystal complex determined at 2.4 Å resolution. Prior to the virtual screening with docking, protein was prepared by using the “Protein Preparation Wizard” workflow in Maestro of the Schrödinger Suite 2010 [30]. The molecular database, SPECS (203,752 compounds, http://www.specs.net), was used as the initial source for screening. These compounds were prepared using LigPrep2.0 [41] to generate low-energy 3D conformations and to determine the ionization states at pH 7.0. Afterward, the default parameters were adopted for two rounds of virtual screening of Glide docking [42], including a high throughput virtual screening (HTVS) and standard precision (SP) docking. After the second round screening, the top 2000 molecules ranked by Gscore were written out together with the receptor in a pose viewer file. At last, the prediction of ligand-receptor binding free energy was performed using MM-GBSA methods provided in the Prime MM-GBSA module [43] in Maestro. The top 500 compounds ranked by MM-GBSA remained for visual analysis to check the potential to form hydrogen bonds (HBs) with protein. Finally, 218 molecules were manually selected and purchased from SPECS for bioassay.

### 3.2. HCV Replicon Assay

Huh7 (NS3-5B) cells correspond to a stable cell line transfected with HCV NS3-5B genotype 1b. The cells were seeded (1 × 104 cells per well) in Dulbecco’s Modified Eagle Medium (DMEM) supplemented with 10% fetal bovine serum, 2 mM glutamine, penicillin (100 IU/mL)/streptomycin (100 μg/mL), 1× nonessential amino acids and 0.5 mg/mL G418 in 96-well plates overnight. Compounds were diluted and added to each well. Each concentration was measured in duplicate. After 48 h of incubation, 100 μL of Steady-Glo Luciferase buffer (E2550, Promega, Beijing, China) was added to each well and shaken for 10 min, and the results were read on plate reader (ENVISION, PerkinElmer, Shanghai, China). The IC_50_ values were calculated by fitting with the parameter of the Hill equation.

### 3.3. Cell Cytotoxicity Assay

To determine if the compounds were cytotoxic to Huh7 (NS3-5B) cells, the cells (1 × 104 cells per well) were plated on 96-well microtiter plates and were incubated at 37 °C in 5% CO_2_ overnight. Various concentrations of the compounds were added to the wells. 48 h later, 10 μL of MTT (M2128, Sigma, Shanghai, China) were added to each well and incubated at 37 °C for 4 h. One-hundred microliters of MTT buffer (10% SDS + 5% isobutyl alcohol + 10 mmol/L HCl) were added overnight, and the optical density readings were measured by colorimeter at 580 and 680 nm.

### 3.4. HCV NS3/4A Protease Assays

A SensoLyte^®^ 490 HCV Protease Assay Kit (AnaSpec, Cat#71126) was used for screening HCV protease inhibitors. The test compounds (100 nL of 200× final concentration, prepared by the ECHO liquid handler (ECHO, Labcyte, CA, USA)) and HCV NS3/4A protease diluent (10 ng, 10 μL) were added into a 384-well black microplate (Corning #3573). The known HCV NS3/4A protease inhibitor (Ac-DEDif-EchaC, AnaSpec Cat#25346) was used as a control. The plate was incubated at room temperature for enzymatic reaction for 10 min. In the meantime, the HCV NS3/4A protease substrate solution was also incubated at the same temperature. The reaction was initiated by adding 10 μL of HCV NS3/4A protease substrate. The reagents were completely mixed by shaking the plate gently for 30–60 s. For the kinetic reading, the fluorescence intensity at *E*x/*E*m = 340 nm/490 nm was immediately measured, and the data was recorded every 2 min for 30 min. The mean (of duplicate wells) inhibition rate at a concentration of 50 μM was measured.

## 4. Conclusions

In summary, this study reports the identification of sixteen novel small molecule inhibitors for hepatitis C virus by structure-based virtual screening of the commercial database, SPECS, in combination with a cellular replicon and NS3/4A protease enzyme assay. The resulting hits exhibited inhibitory activity in the low micro molar range. Among them, compounds **5** and **11** possessed potent antiviral activity with low cytotoxicity, with replicon IC_50_ values of 3.0 μM and 5.1 μM, respectively. Interestingly, these two have low molecular weight and show favorable solubility, which provide new bioactive chemo-types for designing anti-HCV agents.

## Figures and Tables

**Table 1:** *In vitro* data in both cellular replicon and NS3/4A protease enzyme assays of compounds **1**–**10** with the same skeleton selected from virtual screening. CC_50_, the 50% cytotoxicity concentration; MW, molecular weight.

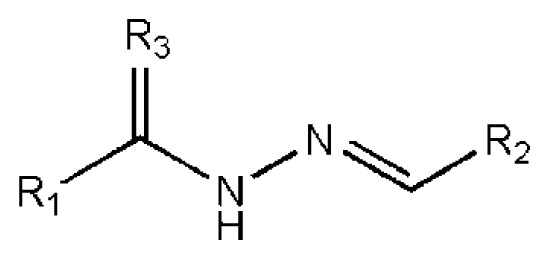										

No.	R_1_	R_2_	R_3_	IC_50_ (μM)	CC_50_ (MTT)	SI a	%Inh. b	MW	Log*P*^c^	Log*S*^c^
**1**	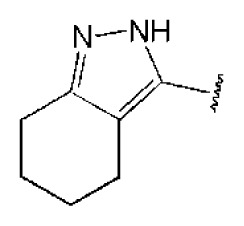	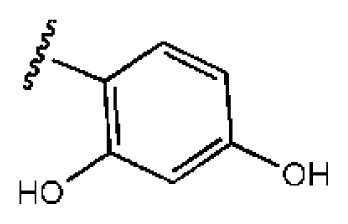	O	4.69	0.46	0.10	96.7	300	0.27	−3.01
**2**	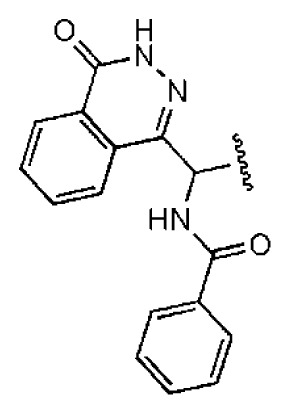	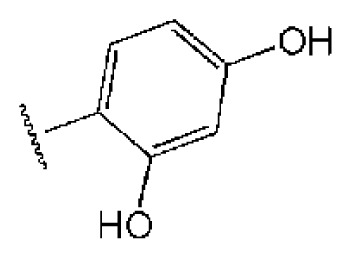	O	11.2	>50	>4.46	77.9	457	1.95	−3.90
**3**	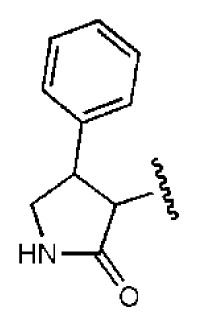	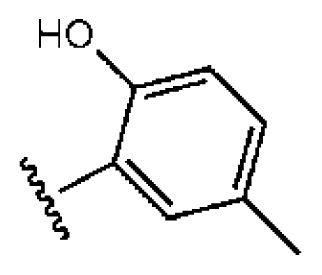	O	13.4	>50	>3.73		337	2.75	−3.69
**4**	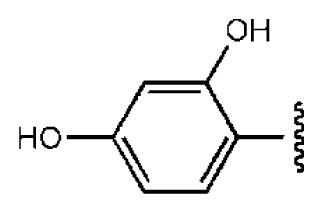	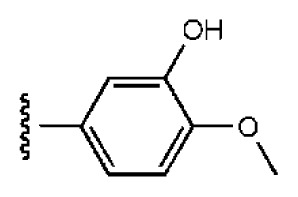	O	15.3	>50	>3.27	56.9	302	2.2	−2.48
**5**	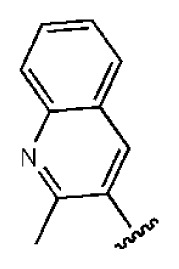	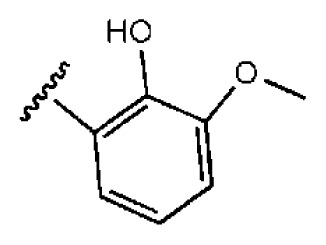	O	3.01	>50	>16.61		335	3.17	−4.05
**6**	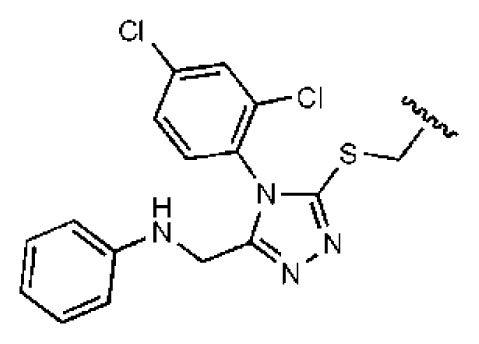	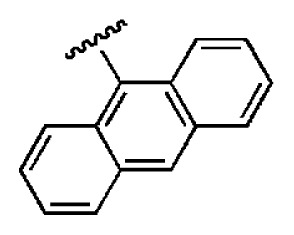	O	5.04	14.8	2.94	48.1	612	7.08	−10.6
**7**	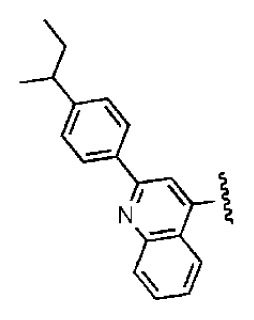	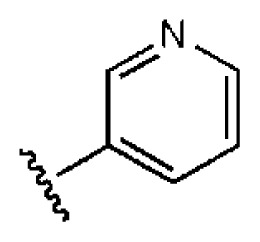	O	4.14	11.8	2.85	64.3	409	5.84	−5.53
**8**	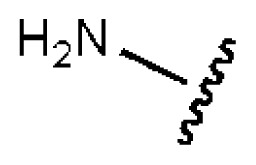	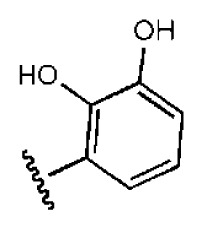	S	6.23	1.12	0.18		211	1.51	−1.89
**9**	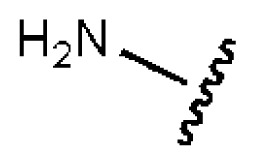	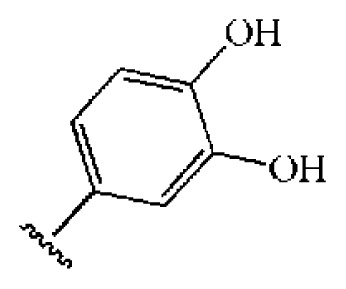	S	21.1	>50	>2.37	33.7	211	1.53	−1.03
**10**	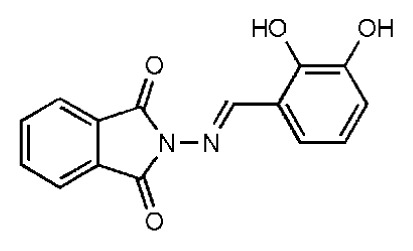			7.07	>50	>7.07		282	2.18	−2.5
**CSA**				0.31	>50	>160				

a Selectivity index (SI): the ratio of CC_50_/IC_50_;

b %Inh.: the inhibition percentage at a concentration of 50 μM in biochemical protease assay;

c Log*P*, Log*S*: the values were provided by SPECS.

**Table 2:** *In vitro* data in both cellular replicon and NS3/4A protease enzyme assays of compounds **11**–**16**.

No.	Structures	IC_50_ (μM)	CC_50_ (MTT)	SI a	%Inh. b	MW	Log*P*^c^	Log*S*^c^
**11**	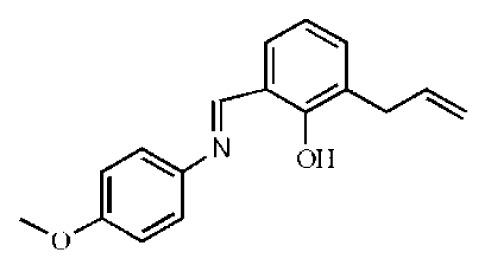	5.14	>50	>9.73		267	4.43	−4.16
**12**	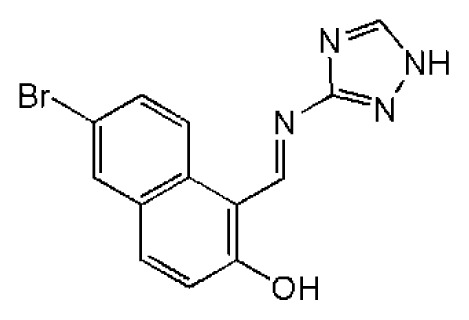	16.1	>50	>3.11	53.0	317	3.23	−2.67
**13**	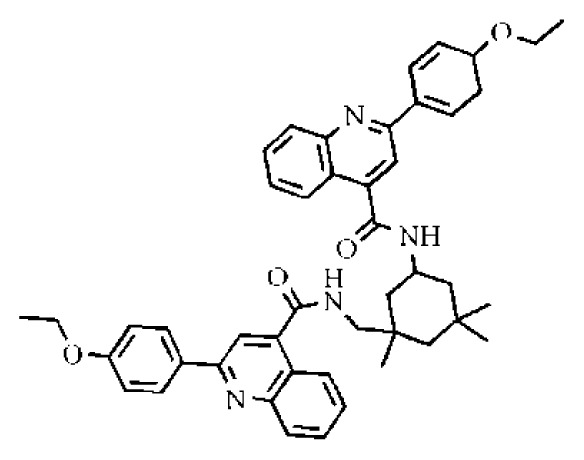	16.3	>50	>3.07		721	10.56	−14.58
**14**	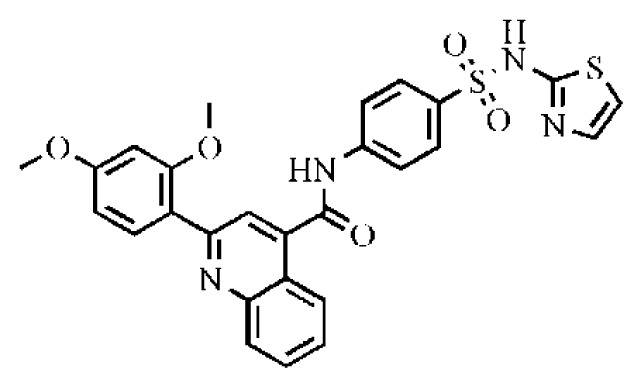	20.7	>50	>2.42		547	5.1	−7.55
**15**	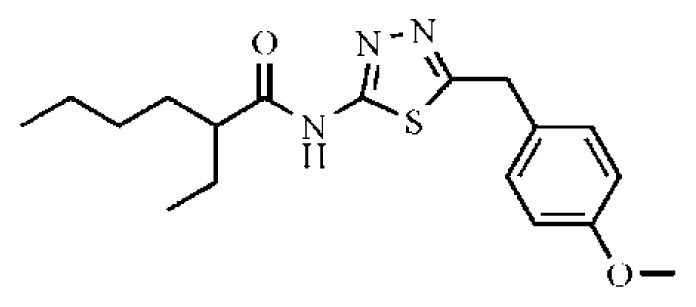	28.2	>50	>1.77	41.8	347	4.57	−5.64
**16**	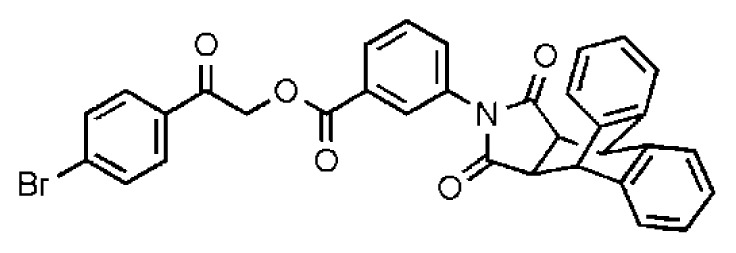	9.42	>50	>5.31		592	4.48	−8.04

a Selectivity index (SI): the ratio of CC_50_/IC_50_;

b %Inh.: the inhibition percentage at a concentration of 50 μM in biochemical protease assay;

c Log*P*, Log*S*: the values were provided by SPECS.
